# IL1β is induced in nephronophthisis but does not mediate kidney damage

**DOI:** 10.1016/j.gendis.2025.101687

**Published:** 2025-05-14

**Authors:** Giulia Ferri, Mariyam El Hamdaoui, Joran Martin, E. Wolfgang Kuehn, Frank Bienaimé, Sophie Saunier, Amandine Viau

**Affiliations:** a Université Paris Cité, Imagine Institute, Laboratory of Hereditary Kidney Diseases, INSERM UMR 1163, Paris F-75015, France; b Renal Division, Department of Medicine, Faculty of Medicine, Medical Center, University of Freiburg, Hugstetter Strasse 55, Freiburg 79106, Germany; c Mechanisms and Therapeutic Strategies of Chronic Kidney Disease, INSERM U1151-CNRS UMR 8253, Université Paris Cité, Institut Necker Enfants Malades, Paris F-75015, France; d Department of Physiology, Necker Hospital, Assistance Publique-Hôpitaux de Paris, Paris F-75015, France

Nephronophthisis (NPH), an autosomal recessive tubulo-interstitial nephropathy, is characterized by interstitial inflammation and progressive kidney fibrosis. To date, mutations in more than 25 *NPHP* genes have been associated with NPH, resulting in a wide genetic heterogeneity and overlapping clinical phenotypes. However, 53% of the patients with a genetic diagnosis have biallelic mutations in *NPHP1*.^1^ Fibrosis is caused by excessive matrix deposition, mainly by activated myofibroblasts. Inflammatory signals play a central role in the differentiation and expansion of myofibroblasts. The interleukin 1 (IL1) family of cytokines is one of the most potent triggers of innate immune response. The activity of IL1 is mediated by its type I receptor (IL1R) whose intracellular domain shares similarities with the Toll protein in fruit flies, known as the Toll interleukin-1 receptor (TIR) domain. When the cytokine binds, IL1R initiates a signaling cascade through recruiting cytoplasmic myeloid differentiation primary response protein 88 (MYD88), IL1R associated kinase 4 (IRAK4) and tumor necrosis factor receptor-associated factor 6 (TRAF6) resulting in the activation of the NFκB pathway, among others. In the kidney, *in vitro* studies reported that both IL1α and IL1β promote TGF-β production and fibronectin production in human proximal tubular cells. IL1β also promoted the conversion of kidney tubular epithelial cells into myofibroblasts, the primary drivers of collagen deposition.^2^ This finding was also supported by another study where treatment with IL1β triggered myofibroblast activation, matrix production, collagen deposition and fibrosis in kidney organoids.^3^

Given the involvement of IL1 dependent signalling in fostering kidney inflammation, immune cell recruitment, and matrix deposition, we hypothesized that IL1 signalling might play a pathogenic role in the context of NPH.

We recently demonstrated that NPH is associated with immune cell infiltration into the kidney.^4^ In NPH-mouse models and urine-derived kidney epithelial cells (UREC) from NPH patients, we showed increased expression of inflammatory chemokines, including CCL2, CX3CL1, CXCL1, and CXCL10, which is associated with infiltration of macrophages, neutrophils, and T cells into the kidney, supporting that inflammation is a central feature of renal ciliopathies. To first seek whether NPH is associated with an increase expression of IL1β, we analyzed UREC of healthy donors or patients bearing *NPHP1* mutations.^1^ We observed that tubular cells derived from *NPHP1* patients displayed increased expression of *IL1B* transcript (Fig. 1A and Table S1–S2). We further confirmed IL1β upregulation in a NPH-like mouse model caused by a bi-allelic mutation of *Lkb1*, a ciliary kinase interacting with NPHP1.^5^ This mouse model allows *Lkb1* deletion specifically in the distal part of the nephrons (*Lkb1*^ΔTub^) recapitulating inflammation and interstitial fibrosis found in NPH patients. We performed quantitative RT-PCR and observed that *Il1β* transcript was significantly increased in *Lkb1*^ΔTub^ kidneys at 5 weeks, an early stage in disease progression,^5^ and further enhanced at 12 weeks compared to control kidneys (Fig. S1). *Il1β* is firstly transcribed as a precursor that is successively cleaved and secreted in its active IL1β form. Thus, we examined the level of expressed protein in kidney lysates from *Lkb1*^ΔTub^ and control mice. ELISA showed that IL1β protein level was higher in *Lkb1*^ΔTub^ than in control kidneys (Fig. S1). To verify whether kidney tubular cells can induce inflammatory response upon IL1β stimulation, we examined whether IL1β treatment increases the expression of specific cytokines deregulated in NPH.^4^ Indeed, most of the cytokines defining NPH inflammatory signature were induced by IL1β treatment (Fig. S1).

**Figure 1:**
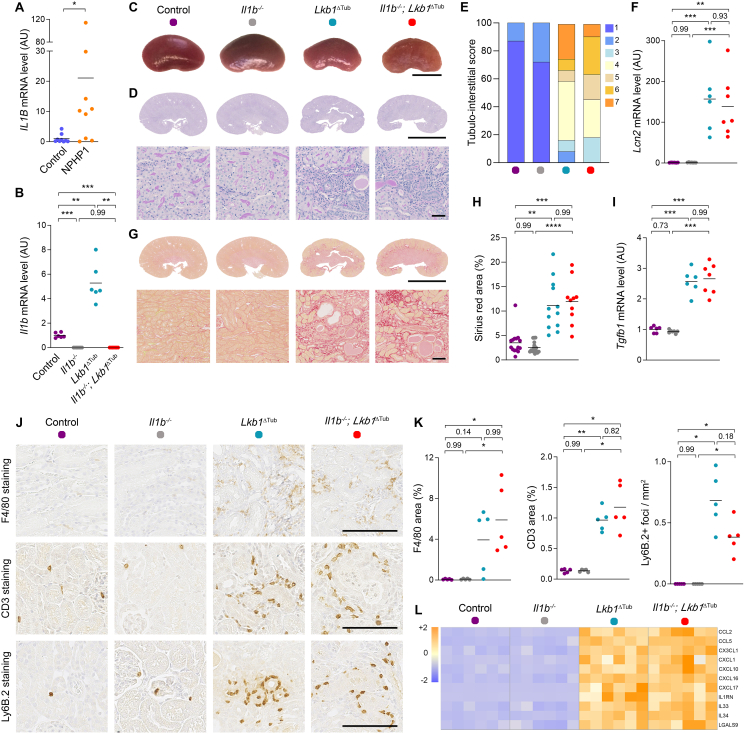
IL1β is fully dispensable for the development of NPH like fibro-inflammatory kidney disease. **(A)***IL1β* mRNA content evaluated by quantitative PCR performed on urine-derived renal epithelial cells (UREC) from controls and *NPHP1* patients. Each dot represents one biological replicate. Mann–Whitney *t* test, ∗*P* < 0.05. **(B)***Il1β* mRNA expression evaluated by qPCR in kidneys from controls, *Il1β*^−/−^, *Lkb1*^ΔTub^ and *Il1β*^−/−^; *Lkb1*^ΔTub^ animals at 12 weeks. ANOVA Brown–Forsythe test with Tamhane's T2 multiple comparison test. AU: arbitrary unit. **(C)** Representative kidney pictures from controls, *Il1β*^−/−^, *Lkb1*^ΔTub^ and *Il1β*^−/−^; *Lkb1*^ΔTub^ mice at 12 weeks. Scale bar: 0.5 cm. **(D)** Representative PAS stained kidney sections from controls, *Il1β*^−/−^, *Lkb1*^ΔTub^ and *Il1β*^−/−^; *Lkb1*^ΔTub^ mice at 12 weeks. Scale bars: 0.5 cm (upper panel) and 50 μm (lower panel). **(E)** Tubulo-interstitial lesion scoring in kidneys from controls, *Il1β*^−/−^, *Lkb1*^ΔTub^ and *Il1β*^−/−^; *Lkb1*^ΔTub^ animals at 12 weeks. The percentage of animals in each scoring category (1–7) is represented. **(F)***Lcn2* mRNA expression evaluated by qPCR in controls, *Il1β*^−/−^, *Lkb1*^ΔTub^ and *Il1β*^−/−^; *Lkb1*^ΔTub^ kidneys at 12 weeks. One-way ANOVA with Tukey's multiple comparison test. **(G**–**H)** Representative Sirius red (G) stained kidney sections and quantification (H) from controls, *Il1β*^−/−^, *Lkb1*^ΔTub^ and *Il1β*^−/−^; *Lkb1*^ΔTub^ mice at 12 weeks. Scale bars: 0.5 cm (upper panel) and 50 μm (lower panel). Kruskal–Wallis test with Dunn's multiple comparison test. **(I)** Pro-fibrotic marker *Tgfb1* mRNA content evaluated by qPCR in kidneys from the same 4 group of mice at 12 weeks. ANOVA Brown–Forsythe test with Tamhane's T2 multiple comparison test. **(J**–**K)** Representative images (J) and quantification (K) of macrophages (F4/80 staining), T cells (CD3 staining) and neutrophils (Ly-6B.2 staining) in kidney sections from 12 week old animals. Scale bar: 100 μm. Kruskal–Wallis test with Dunn's multiple comparison test (F4/80, Ly-6B.2) or ANOVA Brown–Forsythe test with Tamhane's T2 multiple comparison test (CD3). **(L)** Heatmaps showing Z-scores computed on mRNA expression levels measured by quantitative PCR of the indicated pro-inflammatory cytokines in kidneys from 12 week old controls, *Il1β*^−/−^, *Lkb1*^ΔTub^ and *Il1β*^−/−^; *Lkb1*^ΔTub^ mice. See also Figure S3. **(A-B, F, H, I, K)** Each dot represents one individual mouse. Bars indicate mean. ∗*P* < 0.05, ∗∗*P* < 0.01, ∗∗∗∗*P* < 0.0001. AU: arbitrary unit.

To robustly determine if IL1β inhibition would ameliorate NPH-like mouse model, we crossed tubule-specific *Lkb1*^ΔTub^ mice with mice bearing systemic *Il1β* knockout (*Il1b*^−/−^). We compared littermate mice with distal tubular inactivation of *Lkb1* alone (*Lkb1*^ΔTub^), systemic *Il1β* knockout alone (*Il1b*^−/−^) or both (*Il1b*^−/−^; *Lkb1*^ΔTub^). At 12 weeks of age, quantitative RT-PCR showed that, as expected, *Il1b* transcript was not detected in *Il1b*^−/−^ kidneys, while *Il1β* induction in *Lkb1* deficient kidneys was drastically blunted in *Il1b*^−/−^; *Lkb1*^ΔTub^ kidneys (Fig. 1B). Macroscopic inspection revealed that kidneys from *Il1b*^−/−^ resembled to controls supporting the lack of detrimental phenotype in *Il1b*^−/−^. However, kidneys from *Lkb1*^ΔTub^ and *Il1b*^−/−^; *Lkb1*^ΔTub^ showed surface irregularities associated with reduced kidney size, which was even more pronounced in the latter, though not statistically different (Fig. 1C; Fig. S2). Both *Lkb1*^ΔTub^ and *Il1b*^−/−^; *Lkb1*^ΔTub^ mice displayed similar urine concentration defect with increased urinary flow rate and decreased urine osmolality as compared to control and *Il1b*^−/−^ mice (Fig. S2). Kidney failure occurred at a similar range in both *Lkb1*^ΔTub^ and *Il1b*^−/−^; *Lkb1*^ΔTub^ mice as shown by increased levels of blood urea nitrogen (Fig. S2).

*Il1b*^−/−^ animals displayed normal kidney architecture similar to control animals. *Lkb1* tubular depletion led to tubule and glomeruli's basement membranes thickening, tubular atrophies and dilations along with interstitial inflammation irrespective of *Il1b* status (Fig. 1D–E). Consistently, *Il1β* inactivation did not reduce the induction of the tubular injury marker *Lcn2* (Fig. 1F).

We then proceeded with the characterization of interstitial fibrosis in order to evaluate whether depletion of *Il1β* might have an impact on this aspect. PicroSirius red staining and quantification showed an increased level of collagen fibers in kidneys from both *Lkb1*^ΔTub^ and *Il1b*^−/−^; *Lkb1*^ΔTub^ mice, while no statistical difference was observed between control and *Il1b*^−/−^ kidneys (Fig. 1G–H). Increased fibrogenesis was confirmed by measuring fibrosis related transcripts such as *Col1a1*, *Col3a1, Tgfb1* and *Acta2*, all of which were significantly induced in *Lkb1* mutant mice irrespective of *Il1b* status (Fig. 1I; Fig. S2).

Although loss of *Il1β* did not abrogate tubular damage and interstitial fibrosis, we evaluated its impact on kidney inflammation. F4/80 immunostaining revealed a comparable infiltration of mononucleated phagocytes in *Lkb1*^ΔTub^ and *Il1b*^−/−^; *Lkb1*^ΔTub^ kidneys (Fig. 1J–K). In agreement, the mRNA level of *Adgre1*, the gene encoding F4/80, was increased in kidneys from *Lkb1*^ΔTub^ and this was not modified by *Il1β* inactivation (Fig. S3). Stainings for CD3 and LY6B.2 revealed overall a similar pattern with increased T cells and neutrophils recruitment in both *Lkb1*^ΔTub^ and *Il1b*^−/−^; *Lkb1*^ΔTub^ kidneys (Fig. 1J–K). *Lkb1* loss induced NPH pro-inflammatory cytokines, which was not affected by simultaneous *Il1β* deletion (Fig. 1L; Fig. S3). We finally tested if an increased expression of another IL1 family member could have compensated IL1β deficiency. We measured the expression levels of other members of the IL1 family, notably IL1α and IL18. Interestingly, as observed in mouse brain of *Il1b*^−/−^, genetic deletion of *Il1β* drastically reduced the expression of *Il1α* in the kidney (Fig. S3). *Lkb1* loss induced *Il1α* transcript at 12 weeks while *Il1β* deletion almost restored *Il1α* mRNA expression to control level in *Lkb1*^ΔTub^ kidneys. In contrast, *Il1β* loss did not affect IL18 transcription since *Il1β*-deficient mice showed comparable levels to the control group and did not modulate the increased observed in *Lkb1*^ΔTub^ kidneys (Fig. S3).

Altogether, our observations indicate that immune cell recruitment and interstitial fibrosis associated with NPH developed independently of IL1β. This suggests that the upregulation of IL1β occurs as a secondary effect with a marginal role in sustaining inflammation. Therefore, it is crucial to investigate the specific key factors supporting the immune response to develop effective anti-inflammatory therapeutic strategies.

## CRediT authorship contribution statement

**Giulia Ferri:** Writing – original draft, Investigation. **Mariyam El Hamdaoui:** Investigation. **Joran Martin:** Investigation. **E. Wolfgang Kuehn:** Writing – review & editing, Conceptualization. **Frank Bienaimé:** Writing – review & editing, Writing – original draft, Supervision, Investigation, Conceptualization. **Sophie Saunier:** Writing – review & editing, Funding acquisition, Conceptualization. **Amandine Viau:** Writing – review & editing, Writing – original draft, Supervision, Investigation, Formal analysis, Conceptualization.

## Ethics declaration

All animal experiments were conducted according to the guidelines of the National Institutes of Health Guide for the Care and Use of Laboratory Animals, as well as the French laws for animal welfare, and were approved by regional authorities (Ministère de l’Enseignement, de la Recherche et de l’Innovation #26193–2020051216078531).

The NPH_1 protocol on the research of therapeutic targets in the frame of NPH and renal-associated ciliopathies has been approved by the French National Committee for the Protection of Persons under the ID-RCB no. 2016-A00541-50 and is kept in full accordance with the principles of the Declaration of Helsinki and Good Clinical Practice guidelines.

## Funding

Giulia Ferri was supported by the European Union's 10.13039/501100007601Horizon 2020 research and innovation programme under the Marie Sklodowska-Curie Innovative Training Networks SCILS (No. 861329), Joran Martin received a PhD grant from the Ministère de l’Education Nationale, de la Recherche et de la Technologie (MENRT), E. Wolfgang Kuehn was supported by the 10.13039/501100001659Deutsche Forschungsgemeinschaft (DFG, Nos. KU-1504/8-1, KU-1504/9-1, DFG CRC 1453), Frank Bienaimé was supported by the EMBO (No. ALTF927-2013), Sophie Saunier received support from the Institut National de la Santé et de la Recherche Médicale (INSERM), the MENRT and the Agence Nationale de la Recherche (ANR, Nos. ANR-10-IAHU-01, ANR-17-RHUS-0002), Amandine Viau was supported by the ERA-EDTA (No. ALTF84-2011).

## Conflict of interests

The authors declared no competing interests.
