# Supplementary material for: IL1β is induced in nephronophthisis but does not mediate kidney damage
Source: Genes Dis. 2025 May 14;13(2):101687. doi: 10.1016/j.gendis.2025.101687 (PMC12677047; doi:10.1016/j.gendis.2025.101687)

This file includes:

Materials and Methods

Supplementary Figures S1 to S3

Supplementary Tables S1 to S2

**MATERIALS and METHODS**

Isolation of urine-derived renal epithelial cells (UREC)

Urine samples were collected from *NPHP1* patients, healthy relatives and unrelated controls recruited at Necker Hospital (Paris, France) in the frame of the approved NPH_1 protocol upon written informed consent of the donor^1^. The relatives were the healthy relatives (father/mother) of an included patient. Healthy controls were free from any chronic kidney disease or with normal renal function. Patient data are listed in Table S1.

Urine-derived renal tubular epithelial cells (primary UREC) were isolated and cultured as previously described^2^ with some modifications. Briefly, after centrifugation and washing steps, urine-derived cells were initially cultured for 4 days at 37°C in primary medium containing Dulbecco’s Modified Eagle Medium : Nutrient Mixture F-12 (DMEM/F-12) supplemented with 10% fetal bovine serum (16000-036, Gibco), 10% Penicillin-Streptomycin (15140-122, Gibco), 10% Amphotericin B (15290026, Gibco) and 1X REGM^TM^ SingleQuots^TM^ kit (CC-4127, Lonza) to enhance cell survival and adherence. At day 4, primary medium was replaced by a growth medium containing REBM^TM^ (Basal Medium, CC-3191, Lonza) supplemented with 2% fetal bovine serum, 10% Penicillin-Streptomycin, 10% Amphotericin B, 1X REGM^TM^ SingleQuots^TM^ kit and 10 ng/mL rhEGF (R&D system) and was then changed every 2 days. At 80% confluence (7-30 days), 2x10^4^ cells were seeded for 7-24 days until confluence on 12 well plates (353043, Dutscher) for RNA analysis.

Mice

Mice were housed in a specific pathogen-free facility, fed ad libitum and housed at constant ambient temperature in a 12-hour day/night cycle. Breeding and genotyping were done according to standard procedures.

*Lkb1*^ΔTub^ mice were previously described^3^. *Il1b* ^-/-^ mice (C57BL/6) were kindly provided by Flora Zavala (obtained from CDTA, Orléans, France)^4^ and were backcrossed for 2 generations with *Lkb1*^ΔTub^ mice. The progeny was then intercrossed to generate *Il1β* systemic knockout with wild-type *Lkb1* (further referred to as *Il1β* ^-/-^), tubule-specific Lkb1 knockout with wild-type *Il1β* (further referred to as *Lkb1*^ΔTub^) and mice carrying both *Il1β* systemic knockout and tubule-specific Lkb1 knockout (further referred to as *Il1b*^-/-^; *Lkb1*^ΔTub^). Littermates lacking KspCre transgene and carrying wild-type *Il1β*  were used as controls. Experiments were conducted on both females and males.

Urine and Plasma Analyses

One week before sacrifice, mice were placed in metabolic cages with access to water and food and urine was collected for 24 hours. Urine osmolality was measured with a freezing point depression osmometer (Micro-Osmometer from Knauer). Urine excretion was measured.

The day of sacrifice, retro-orbital blood was collected from anesthetized mice. Plasma blood urea nitrogen (BUN) was measured using a Konelab 20i Analyzer (Thermo Scientific).

Cell culture

Mouse Inner Medullary Collecting Duct cells (mIMCD3, ATCC) were cultured using Dulbecco's Modified Eagle Medium/Nutrient Mixture F-12 (GIBCO, 21331-020) supplemented with 10% fetal bovine serum, 1% L-glutamine (GIBCO, 15140-122) and 1% penicillin–streptomycin (GIBCO, 25030-024). 50,000 cells/cm² were seeded on 12 well plate (Falcon, 353043) and after 3 days of culture cells were overnight serum starved. The following day, cells were treated for 6 hours with 10ng/ml of mouse IL1β (Sigma, I5271) or vehicle. Then, RNA was extracted and qRT–PCR were performed. All cells were regularly tested for mycoplasma contamination and were mycoplasma-free.

Quantitative RT-PCR

Total RNAs were obtained from cells of female mouse kidneys using RNeasy Mini Kit (Qiagen) and reverse transcribed using High Capacity cDNA Reverse Transcription Kit (Applied Biosystems) according to the manufacturer’s protocol. Quantitative PCR were performed with iTaq™ Universal SYBR® Green Supermix (Bio-Rad) on a CFX384 C1000 Touch (Bio-Rad). *Gapdh*, *Hprt, Ppia* and *Rpl13* were used as normalization controls^5^.Each biological replicate was measured in technical duplicates. The primers used for qRT-PCR are listed in Supplementary Table 2. Heatmap displaying Z-scores computed on the expression levels of the identified cytokines, measured by qPCR, was generated using excel.

Morphological Analysis

Mouse kidneys were fixed in 4% paraformaldehyde, embedded in paraffin, and 4µm sections were stained with periodic acid-Schiff (PAS) or Picrosirius Red. Stained full size images were recorded using a whole slide scanner Nanozoomer 2.0 (Hamamatsu) equipped with a 20x/0.75 NA objective coupled to NDPview software (Hamamatsu). Tubulo-interstitial score was evaluated by two independent observers in a blinded fashion assessing the overall lesions of the whole kidney section stained with PAS. Seven scores were defined ranging from score 1 normal kidney architecture to score 7 associating tubular atrophy, tubular basement thickening and interstitial cell infiltration. For fibrosis quantification, PicroSirius Red stained area was measured with ImageJ software from full sized kidney images and visualized as the percentage of stained surface to total kidney section area.

Immunohistochemistry

Four-micrometer sections of paraffin-embedded female kidneys were submitted to antigen retrieval and avidin/biotin blocking (Vector, SP-2001). Sections were incubated with primary antibodies as followed: CD3 (Abcam, ab16669, 1:100), F4/80 (Clone Cl:A3-1, Bio-Rad, MCA497R, 1:100) and Ly-6B.2 (Abcam, ab53457, 1:100). Sections were then incubated with biotinylated antibodies (Horse anti-rabbit Vector Lab BA-1100, Rabbit anti-rat biotinylated Vector BA-4001) and HRP-labeled streptavidin (Southern Biotech, 7100-05, 1:2,000). Sections were finally submitted to 3,30-diaminobenzidine-tetrahydrochloride (DAB) revelation.

Full size images were recorded using a whole slide scanner Nanozoomer 2.0 (Hamamatsu) coupled to NDPview software. For macrophage (F4/80 staining) and T cells (CD3 staining) quantification, stained area was measured with ImageJ software from full sized kidney images and visualized as the percentage of stained DAB surface to total kidney section area. For neutrophil (LY6B.2 staining) quantification, since neutrophils are rare and focal, we counted manually the number of foci in whole kidney section. The number of foci per kidney section was scaled to the surface of the section (mm^2^). Foci were defined as 4 or more neutrophils surrounding a single tubule. For all quantification, glomerular and non-specific intra-tubular staining were removed from the analysis.

IL1β ELISA

For the quantification of renal IL1β, kidneys were perfused via the renal artery with PBS1X and homogenized with Cell Lysis Buffer 2 (R&D systems, 845347) according to manufacturer’s procedure. IL1β levels in kidney lysates were immediately quantified using Mouse IL-1 beta/IL-1F2 Quantikine® ELISA Kit (R&D system, MLB00C) according to the manufacturer’s instructions. The samples were used without dilution and were tested in duplicates. The optical densities were derived from 4-parameter logistic regression of the standard curve. Measurement of protein concentration in kidney lysates was performed in the same samples using BCA Protein Assay Kit (Pierce, 23227). The ELISA results were normalized to the protein concentration.

Statistical analysis

Data were expressed as means. Shapiro-Wilk test was performed to verify the distribution of the data. For data that did not follow a normal distribution, differences between groups were evaluated using Student’s Mann-Whitney test (when only two groups were compared) or Kruskal-Wallis test with Dunn’s multiple comparison test (when testing more comparisons). For data that followed normal distribution, Student’s unpaired *t* test with Welch’s correction was used when comparing only two sets of data. When testing more comparisons, One-way ANOVA with Tukey’s multiple comparison test or ANOVA Brown-Forsythe test with Tamhane’s T2 multiple comparison test were used depending on variance differences. The statistical analysis was performed using GraphPad Prism V8 software. All image analyses and mouse phenotypic analyses were performed in a blinded fashion.

Study approval

All animal experiments were conducted according to the guidelines of the National Institutes of Health Guide for the Care and Use of Laboratory Animals, as well as the French laws for animal welfare, and were approved by regional authorities (Ministère de l’Enseignement, de la Recherche et de l’Innovation #26193-2020051216078531).

The NPH_1 protocol on the research of therapeutic targets in the frame of NPH and renal-associated ciliopathies has been approved by the French National Committee for the Protection of Persons under the ID-RCB no. 2016-A00541-50 and is kept in full accordance with the principles of the Declaration of Helsinki and Good Clinical Practice guidelines.

**REFERENCES**

1. Garcia, H. *et al.* Agonists of prostaglandin E2 receptors as potential first in class treatment for nephronophthisis and related ciliopathies. *Proc. Natl. Acad. Sci. U. S. A.* **119**, (2022).

2. Ajzenberg, H. *et al.* Non-invasive sources of cells with primary cilia from pediatric and adult patients. *Cilia* **4**, (2015).

3. Viau, A. *et al.* Cilia-localized LKB1 regulates chemokine signaling, macrophage recruitment, and tissue homeostasis in the kidney. *EMBO J.* **37**, 1–21 (2018).

4. Korniotis, S. *et al.* Mobilized Multipotent Hematopoietic Progenitors Stabilize and Expand Regulatory T Cells to Protect Against Autoimmune Encephalomyelitis. *Front. Immunol.* **11**, (2020).

5. Vandesompele, J. *et al.* Accurate normalization of real-time quantitative RT-PCR data by geometric averaging of multiple internal control genes. *Genome Biol.* **3**, 1–12 (2002).

**
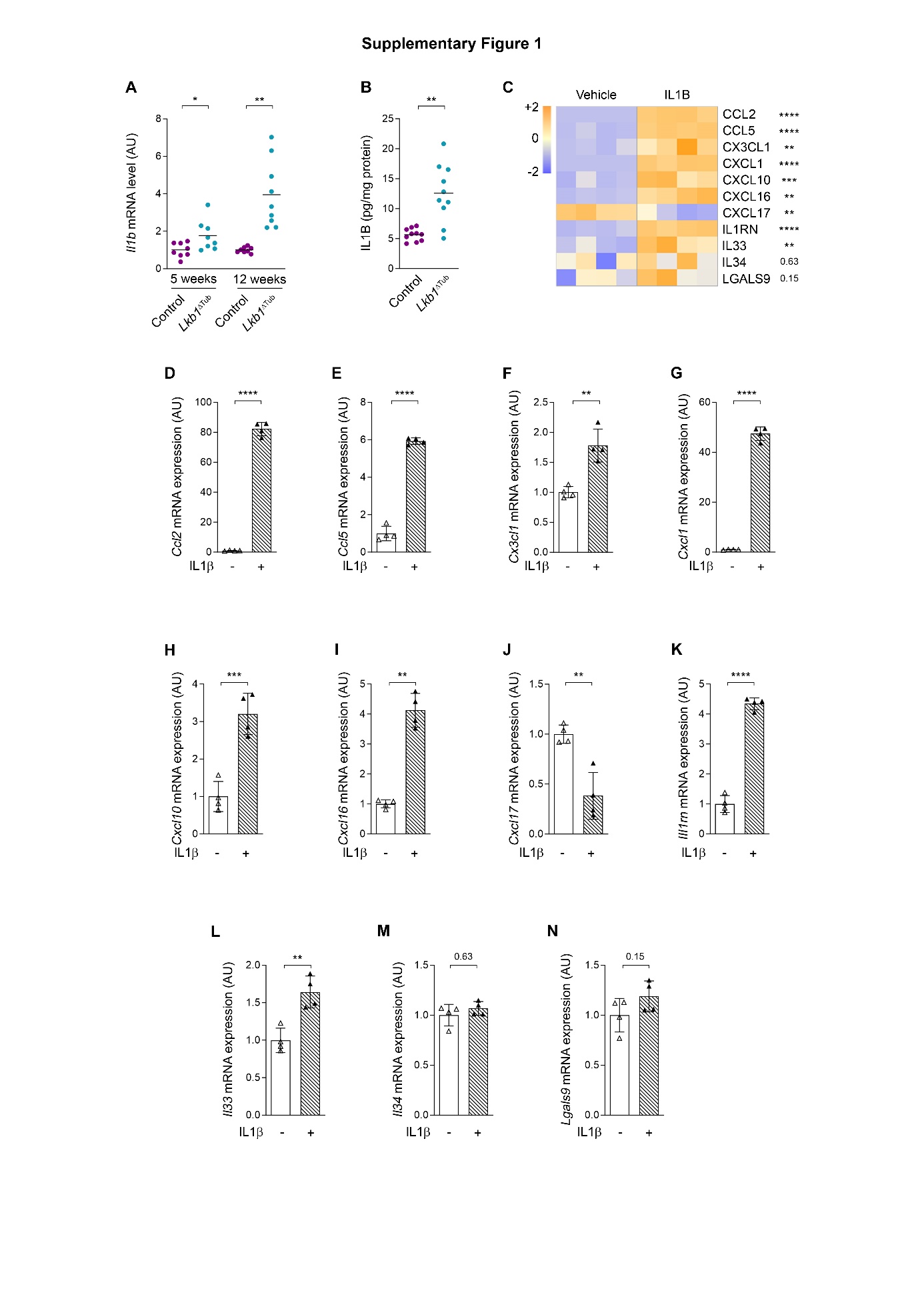
SUPPLEMENTARY FIGURES**

**Figure S1**. Loss of tubular *Lkb1* induces IL1β* *upregulation and secretion. **(A)** Quantitative PCR evaluation of *Il1β* mRNA in kidneys from controls and *Lkb1*^ΔTub^ mice at 5 and 12 weeks of age. Each dot represents one individual animal. Unpaired *t* test, * P < 0.05, ** P < 0.01. **(B)** IL1β protein levels assayed by ELISA in kidneys from controls and *Lkb1*^ΔTub^ mice at 8 weeks. Each dot represents one individual animal. Unpaired *t* test, ** P < 0.01. **(C-N)** Heatmap (C) generated from z-scored obtained from quantitative PCR (D-N) of *Ccl2, Ccl5, Cx3cl1, Cxcl1, Cxcl10, Cxcl16, Cxcl17, Il1rn, Il33, Il34, Lgals9* mRNA levelsof mIMCD3 cells stimulated for 6 hours with IL1β or vehicle. Unpaired *t* test, * P < 0.05, ** P < 0.01, *** P < 0.001, **** P < 0.0001. **(A-B, D-N)** Bars indicate mean. AU: arbitrary unit.


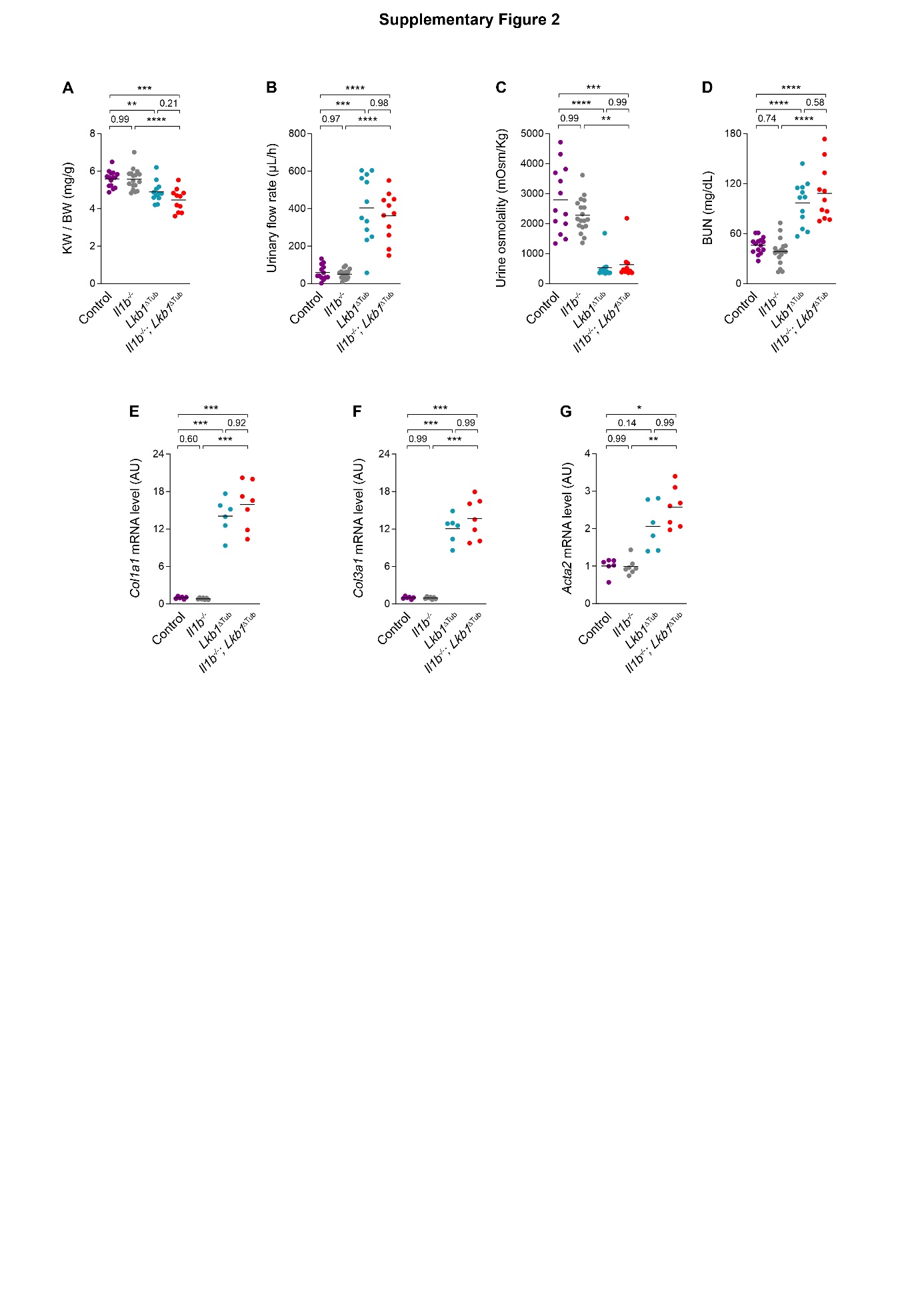


**Figure S2**. Concomitant inactivation of *Il1*β does not affect kidney function decline nor reduce fibrosis after *Lkb1* inactivation. **(A)** Kidney weight (KW) to body weight (BW) ratio in the 4 groups of animals at 12 weeks. One-way ANOVA with Tukey’s multiple comparison test. **(B)** Urinary flow rate calculated through 24 hours urine collection in the 4 groups of mice at 12 weeks. ANOVA Brown-Forsythe test with Tamhane’s T2 multiple comparison test. **(C)** Urine osmolality in the 4 groups of mice at 12 weeks. Kruskal-Wallis test with Dunn’s multiple comparison test. **(D)** Plasma blood urea nitrogen (BUN) in controls, *Il1β* ^-/-^, *Lkb1*^ΔTub^ and *Il1β* ^-/-^; *Lkb1*^ΔTub^ miceat 12 weeks. ANOVA Brown-Forsythe test with Tamhane’s T2 multiple comparison test. **(E-F)** Kidney collagen content evaluated by qPCR measuring *Col1a1* (E) and *Col3a1* (F) mRNA expression in controls, *Il1β* ^-/-^, *Lkb1*^ΔTub^ and *Il1β* ^-/-^; *Lkb1*^ΔTub^ kidneys at 12 weeks. ANOVA Brown-Forsythe test with Tamhane’s T2 multiple comparison test. **(G)** Pro-fibrotic markers *Acta2* mRNA content evaluated by qPCR in kidneys from the same 4 group of mice at 12 weeks. Kruskal-Wallis test with Dunn’s multiple comparison test. **(A-G)** Each dot represents one individual mouse. Bars indicate mean. * P < 0.05, ** P < 0.01, *** P < 0.001, **** P < 0.0001.


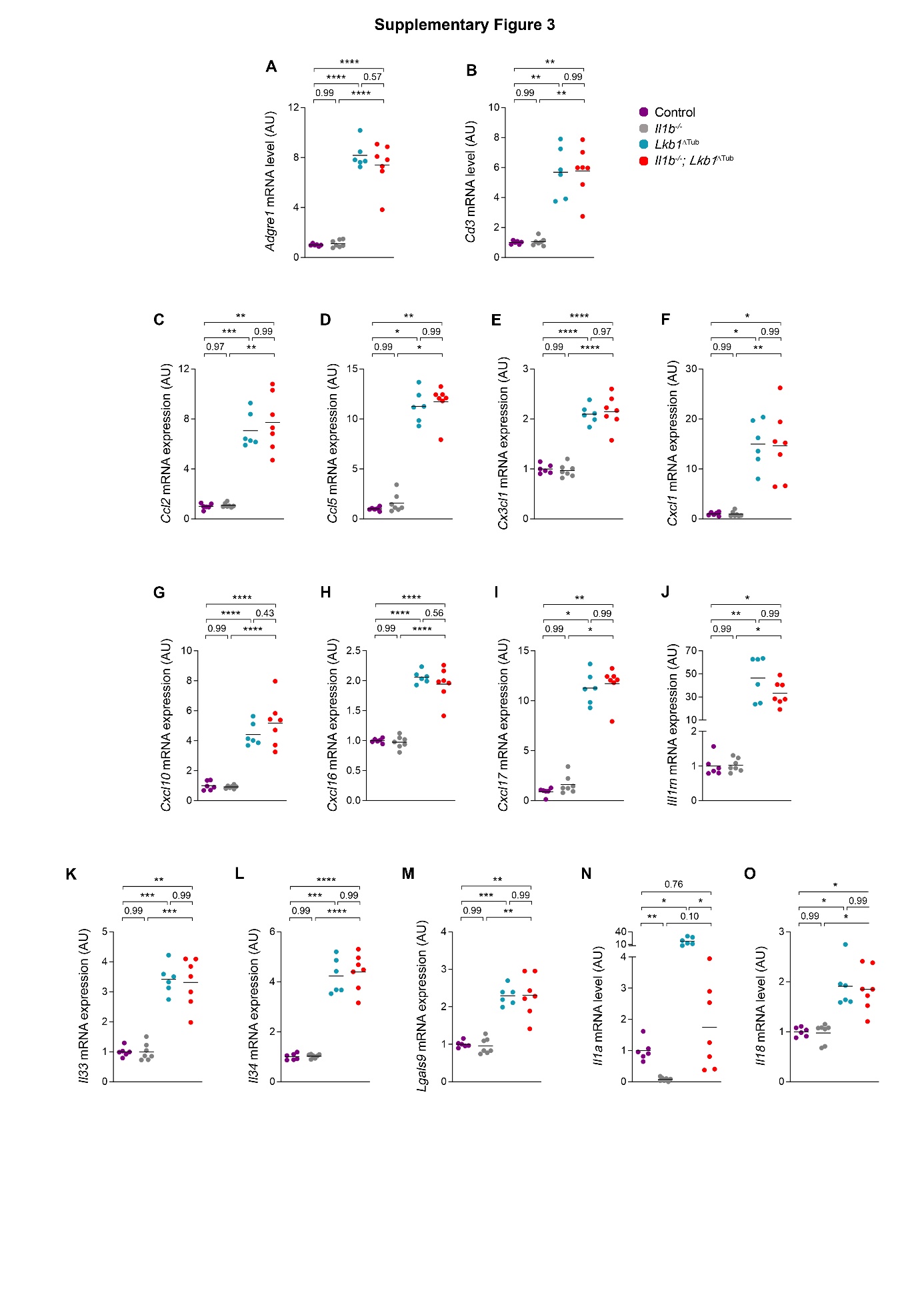


**Figure S3**. *Il1β* inactivation does not reduce the infiltration of *Lkb1* deficient kidneys by immune cells. **(A)** *Adgre1* mRNA expression in controls, *Il1β* ^-/-^, *Lkb1*^ΔTub^ and *Il1β* ^-/-^; *Lkb1*^ΔTub^ kidneys at 12 weeks. One-way ANOVA with Tukey’s multiple comparison test. **(B)** *Cd3* mRNA expression in controls, *Il1β* ^-/-^, *Lkb1*^ΔTub^ and *Il1β* ^-/-^; *Lkb1*^ΔTub^ kidneys at 12 weeks. ANOVA Brown-Forsythe test with Tamhane’s T2 multiple comparison test. **(C-M)** *Ccl2, Ccl5, Cx3cl1, Cxcl1, Cxcl10, Cxcl16, Cxcl17, Il1rn, Il33, Il34, Lgals9* mRNA content evaluated by quantitative PCR in controls, *Il1β* ^-/-^, *Lkb1*^ΔTub^ and *Il1β*^-/-^; *Lkb1*^ΔTub^ kidneys at 12 weeks. **(N)** *Il1α* mRNA content evaluated by qPCR in controls, *Il1β* ^-/-^, *Lkb1*^ΔTub^ and *Il1b*^-/-^; *Lkb1*^ΔTub^ kidneys at 12 weeks. ANOVA Brown-Forsythe test with Tamhane’s T2 multiple comparison test. **(O)** *Il18* mRNA expression evaluated by qPCR in kidneys from controls, *Il1β* ^-/-^, *Lkb1*^ΔTub^ and *Il1β* ^-/-^; *Lkb1*^ΔTub^ mice at 12 weeks. Kruskal-Wallis test with Dunn’s multiple comparison test. Each dot represents one individual mouse. Bars indicate mean. * P < 0.05, ** P < 0.01, *** P < 0.001, **** P < 0.0001. AU: arbitrary unit.

**
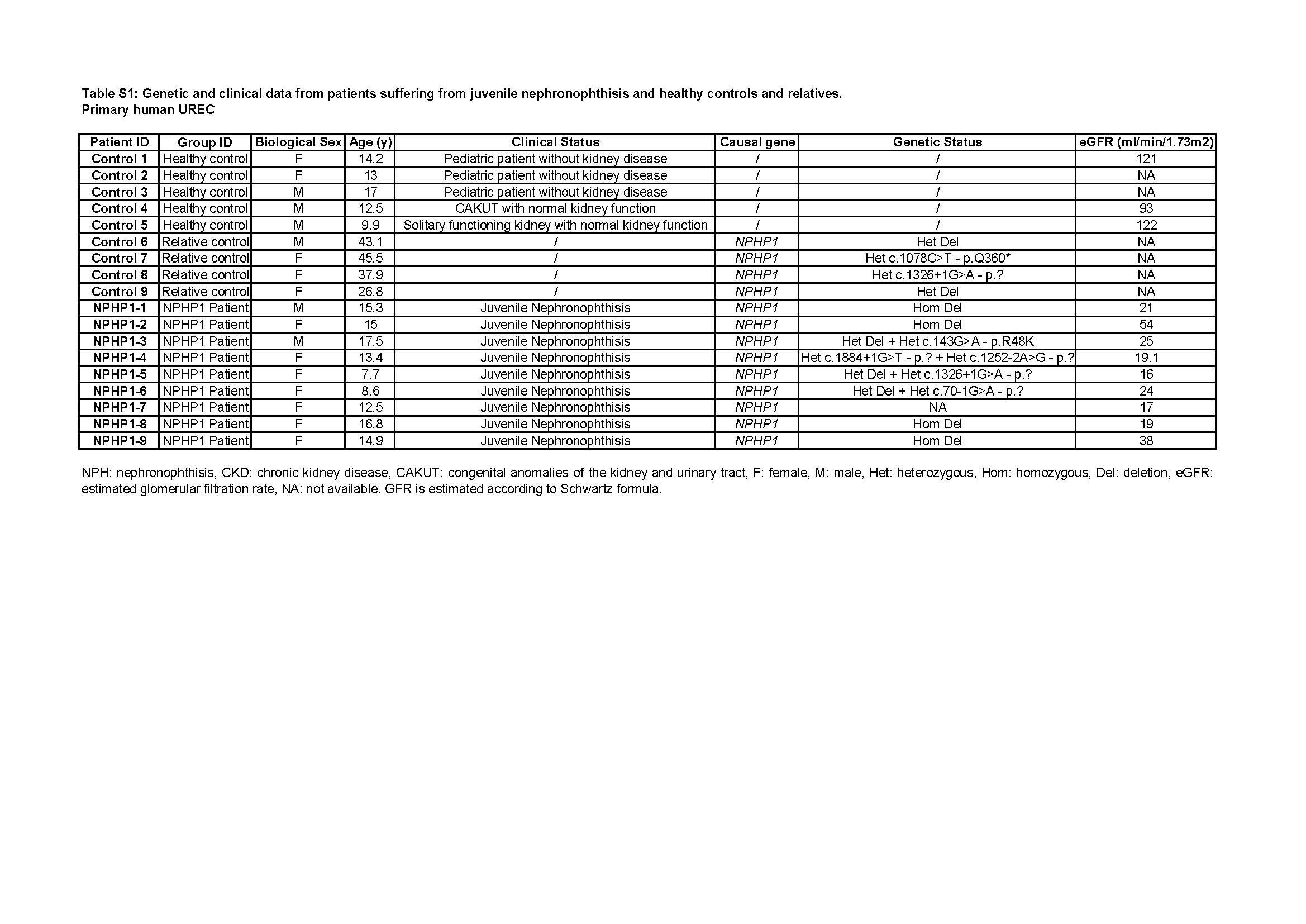
Supplementary Tables**


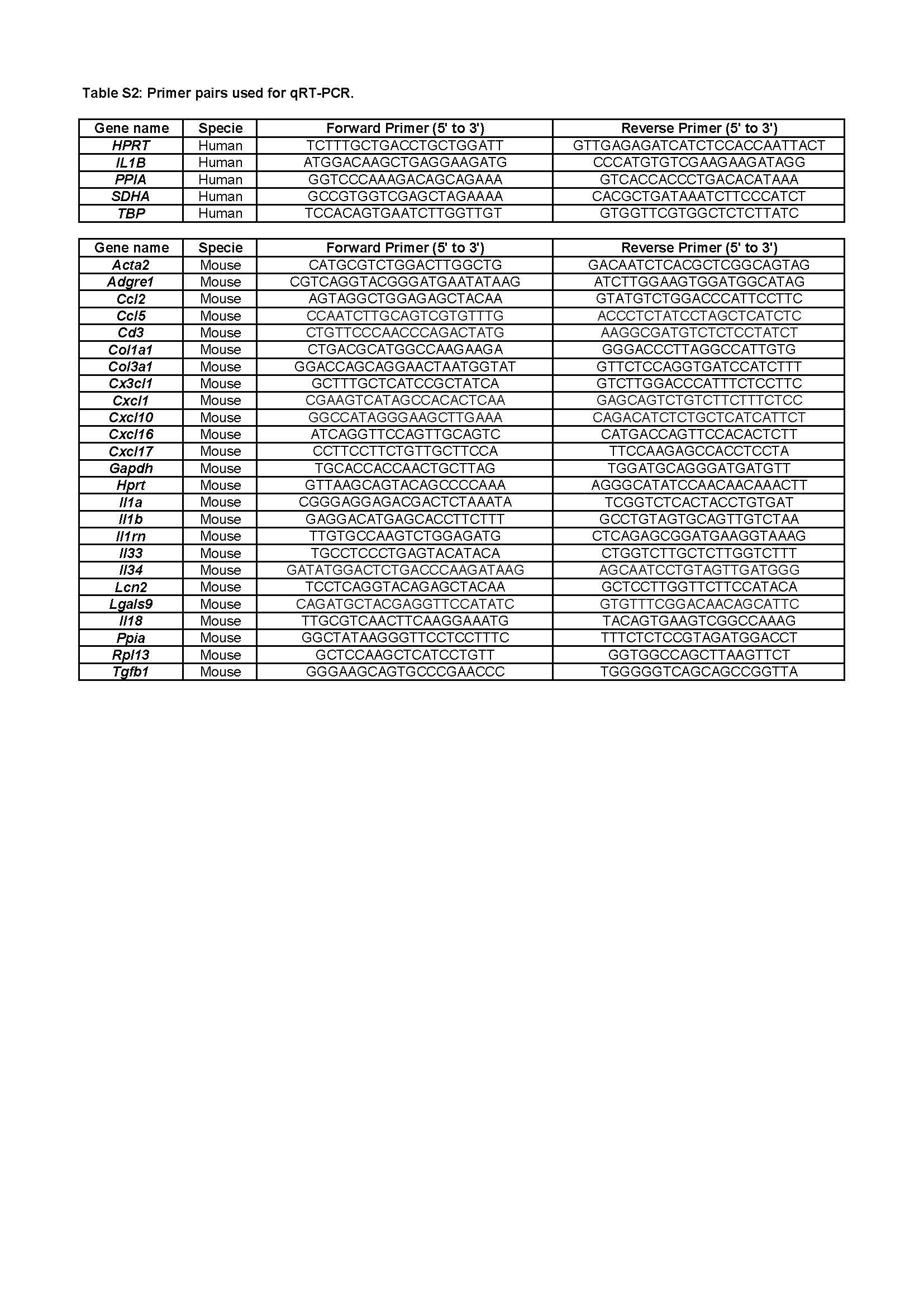

Supplement: Multimedia component 1 [file mmc1.docx]
